# Supplementary material for: Redirecting electron flow in Acetobacterium woodii enables growth on CO and improves growth on formate
Source: Nat Commun. 2024 Jun 26;15:5424. doi: 10.1038/s41467-024-49680-5 (PMC11208171; doi:10.1038/s41467-024-49680-5)
Supplement: Supplementary file 1 — Supplementary Information [file 41467_2024_49680_MOESM1_ESM.pdf]

Redirecting electron flow in *Acetobacterium woodii* enables growth on CO and improves growth on formate

Moon *et al.*

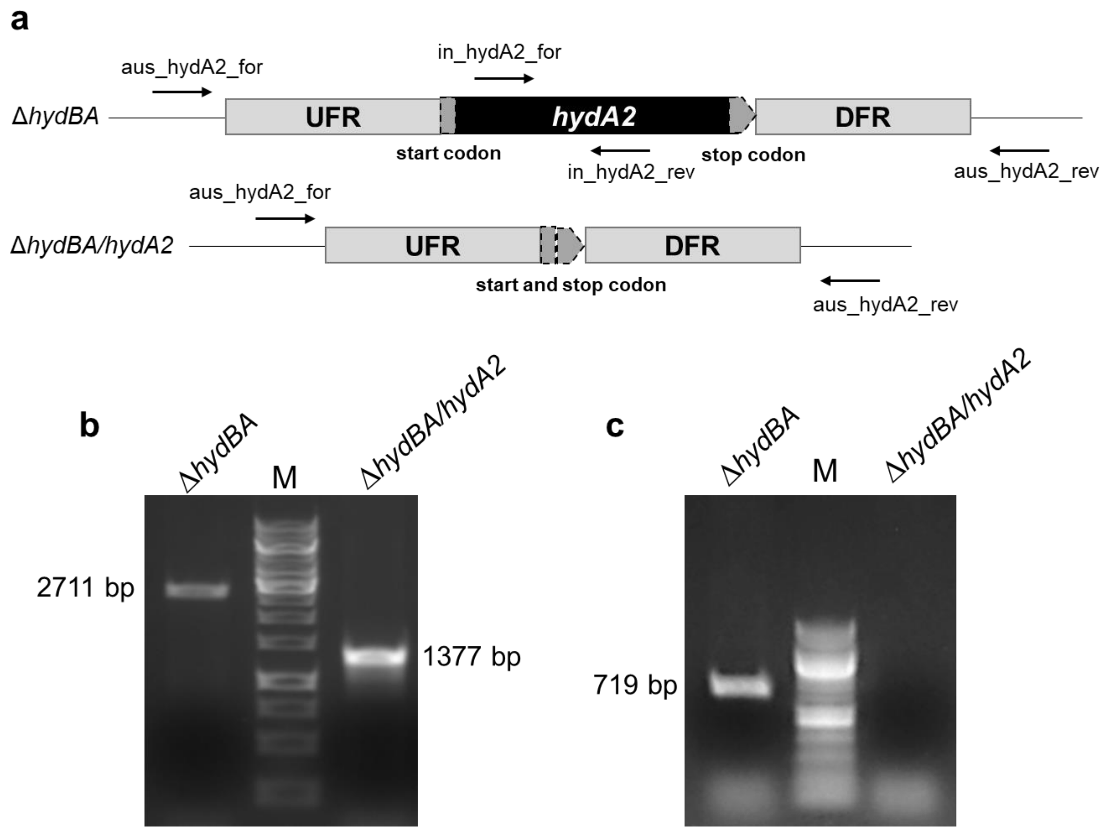

Supplementary Fig. 1. Deletion of the *hydA2* genes in the chromosome of the Δ*hydBA* mutant. (a) The suicide plasmid pMTL_84151_JM_dhydA2 has each 500 bp of upstream- and downstream flanking region (UFR and DFR) of the *hydA2* gene, leaving only the start and stop codon. Furthermore, the plasmid has the *catP* gene from *C. perfringens* for chloramphenicol/thiamphenicol resistance[1] and the *pyrE* gene from *E. limosum* for counter selection using uracil auxotrophy[2]. First, the plasmid was integrated into the chromosome of the Δ*hydBA* mutant by homologous recombination at one flanking region under antibiotic pressure with thiamphenicol and subsequently, disintegration was forced by counter selection with 5-fluoroorotic acid. Cells were grown on fructose and formate, a substrate combination shown previously to allow growth of the Δ*hydBA* and the Δ*hdcr* mutant[2,3]. The deletion of the *hydA2* gene was confirmed by PCR experiments with primers binding outside the deleted region (b) (aus_hydA2_for and aus_hydA2_rev) and by Sanger sequencing[4]. As expected, the *hydA2* gene could not be amplified with primers binding inside of *hydA2* (c) (in_hydA2_for and in_hydA2_rev). Source data are provided as a Source Data file.

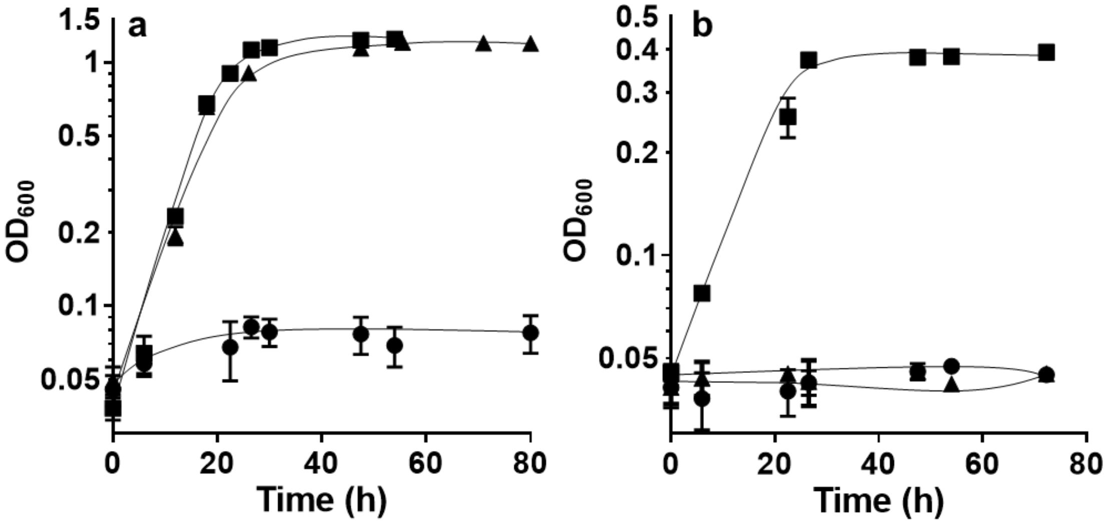

Supplementary Fig. 2. The Δ*hydBA/hydA2* mutant of *A. woodii* grows on fructose. Growth experiments were carried out in 5 ml bicarbonate-buffered complex medium in 16 mL Hungate tubes under a N_2/CO_2 atmosphere (80:20, v/v) at 30 °C with the Δ*pyrE* (squares), Δ*hydBA/hdcr* (circles), and the Δ*hydBA/hydA2* mutant (triangles). Precultures were grown either on 5 mM fructose (Δ*pyrE*) or 5 mM fructose and 5 mM formate (Δ*hydBA/hdcr* and Δ*hydBA/hydA2*) and transferred with a 5% inoculum size to media containing 20 mM fructose (a) or H_2 and CO_2 (2 bar, 80:20, v/v) (b). Each data point presents a mean ± standard deviation (SD); $n = 3$ independent experiments. Source data are provided as a Source Data file.

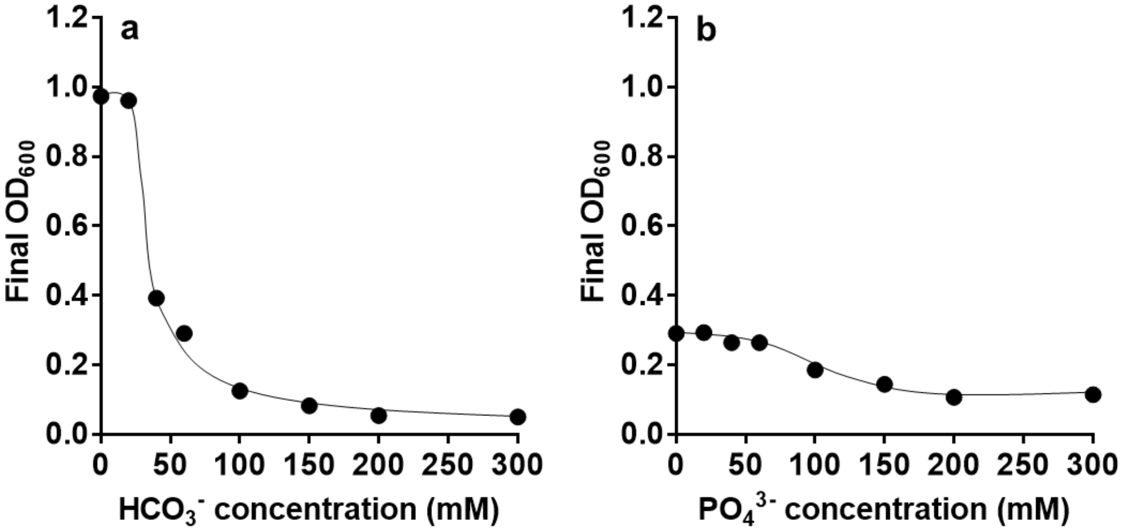

Supplementary Fig. 3. Effect of bicarbonate and phosphate on growth of the Δ*hydBA/hydA2* mutant on formate. Cells of the CO-adapted Δ*hydBA/hydA2* mutant were grown on 100 mM formate either in phosphate-buffered (60 mM) complex medium with various concentrations of potassium bicarbonate (a) or in bicarbonate-buffered (60 mM) complex medium with various concentrations of potassium phosphate (pH 7.0) (b) under a N_2/CO_2 atmosphere (80:20, v/v). Each data point presents a mean ± SD; $n = 2$ independent experiments. Source data are provided as a Source Data file.

Supplementary references

1. Werner, H., Krasemann, C., Gorniak, W., Hermann, A. & Ungerechts, J. Die thiamphenicol- und chloramphenicol-empfindlichkeit von anaerobiern. *Zentralbl. Bakteriol. Orig. A.* **237**, 358-371 (1977).
2. Wiechmann, A., Ciurus, S., Oswald, F., Seiler, V.N. & Müller, V. It does not always take two to tango: "Syntrophy" *via* hydrogen cycling in one bacterial cell. *ISME J.* **14**, 1561-1570 (2020).
3. Moon, J., Schubert, A., Waschinger, L.M. & Müller, V. Reprogramming the metabolism of an acetogenic bacterium to homoformatogenesis. *ISME J.* **17**, 984-992 (2023).
4. Sanger, F.S., Nickelen, F. & Coulson, A.R. DNA-sequencing with chain-terminating inhibitors. *Proc. Natl. Acad. Sci. U.S.A.* **74**, 5463-5467 (1977).
