# Supplementary material for: Redirecting electron flow in Acetobacterium woodii enables growth on CO and improves growth on formate
Source: Nat Commun. 2024 Jun 26;15:5424. doi: 10.1038/s41467-024-49680-5 (PMC11208171; doi:10.1038/s41467-024-49680-5)
Supplement: Supplementary file 3 — Reporting Summary [file 41467_2024_49680_MOESM3_ESM.pdf]

nature portfolio

Corresponding author(s): Prof. Dr. Volker Müller

Last updated by author(s): Jun 10, 2024

Reporting Summary

Nature Portfolio wishes to improve the reproducibility of the work that we publish. This form provides structure for consistency and transparency in reporting. For further information on Nature Portfolio policies, see our Editorial Policies and the Editorial Policy Checklist.

Statistics

For all statistical analyses, confirm that the following items are present in the figure legend, table legend, main text, or Methods section.

n/a	Confirmed	
☐	☒	The exact sample size (n) for each experimental group/condition, given as a discrete number and unit of measurement
☐	☒	A statement on whether measurements were taken from distinct samples or whether the same sample was measured repeatedly
☒	☐	The statistical test(s) used AND whether they are one- or two-sided<br>*Only common tests should be described solely by name; describe more complex techniques in the Methods section.*
☒	☐	A description of all covariates tested
☒	☐	A description of any assumptions or corrections, such as tests of normality and adjustment for multiple comparisons
☐	☒	A full description of the statistical parameters including central tendency (e.g. means) or other basic estimates (e.g. regression coefficient) AND variation (e.g. standard deviation) or associated estimates of uncertainty (e.g. confidence intervals)
☒	☐	For null hypothesis testing, the test statistic (e.g. F, t, r) with confidence intervals, effect sizes, degrees of freedom and P value noted<br>*Give P values as exact values whenever suitable.*
☒	☐	For Bayesian analysis, information on the choice of priors and Markov chain Monte Carlo settings
☒	☐	For hierarchical and complex designs, identification of the appropriate level for tests and full reporting of outcomes
☒	☐	Estimates of effect sizes (e.g. Cohen's d, Pearson's r), indicating how they were calculated

Our web collection on statistics for biologists contains articles on many of the points above.

Software and code

Policy information about availability of computer code

Data collection	WinASPECT v.2.5.0.0 was used for collecting photometric data. TotalChrom Navigator v.6.3.2.0646 was used for collecting gas chromatography data. Chromeleon v.6.8 was used for collecting high performance liquid chromatography data. Trimmomatic v.0.39 was used to trim Ilumina raw reads. Bowtie2 v.2.4.1 was used for mapping against the reference genome. Samtools v.1.9 was used for pileup file for SNP calling.
Data analysis	WinASPECT v.2.5.0.0 was used for analyzing photometric data. TotalChrom Navigator v.6.3.2.0646 was used for analyzing gas chromatography data. Chromeleon v.6.8 was used for analyzing high performance liquid chromatography data. Breseq v.0.32.0 was used for detection of SNPs.

For manuscripts utilizing custom algorithms or software that are central to the research but not yet described in published literature, software must be made available to editors and reviewers. We strongly encourage code deposition in a community repository (e.g. GitHub). See the Nature Portfolio guidelines for submitting code & software for further information.

Data

Policy information about availability of data

All manuscripts must include a data availability statement. This statement should provide the following information, where applicable:

- Accession codes, unique identifiers, or web links for publicly available datasets
- A description of any restrictions on data availability
- For clinical datasets or third party data, please ensure that the statement adheres to our policy

The SNP-sequencing generated during this study is available in the Sequence Read Archive (SRA) with following accession codes: SRR28714882 (https://www.ncbi.nlm.nih.gov/sra/?term=SRR28714882), SRR28714883 (https://www.ncbi.nlm.nih.gov/sra/?term=SRR28714883) and SRR28714884 (https://www.ncbi.nlm.nih.gov/sra/?term=SRR28714884). Source data are provided with this paper.

Research involving human participants, their data, or biological material

Policy information about studies with human participants or human data. See also policy information about sex, gender (identity/presentation), and sexual orientation and race, ethnicity and racism.

Reporting on sex and gender	N/A
Reporting on race, ethnicity, or other socially relevant groupings	N/A
Population characteristics	N/A
Recruitment	N/A
Ethics oversight	N/A

Note that full information on the approval of the study protocol must also be provided in the manuscript.

Field-specific reporting

Please select the one below that is the best fit for your research. If you are not sure, read the appropriate sections before making your selection.

☒ Life sciences ☐ Behavioural & social sciences ☐ Ecological, evolutionary & environmental sciences

For a reference copy of the document with all sections, see nature.com/documents/nr-reporting-summary-flat.pdf

Life sciences study design

All studies must disclose on these points even when the disclosure is negative.

Sample size	No sample size calculation was performed but a sample size of two or three independent biological replicates were used for in vitro analysis to provide data sets that enable statistics for the relevant experiments. The in vitro experiments are tried-and-tested procedures that have been successfully performed in the author's laboratories day-to-day for many years. The sample sizes were diligently chosen and accurately illustrate the differences throughout the various conditions.
Data exclusions	No data were excluded from the anlysis.
Replication	The exact number of replicates is stated in the relevant figure legend. All attempts at replicating particular results were successful.
Randomization	Randomization is not relevant to this study because the samples from defined groups.
Blinding	Blinding is not relevant to this study since no higher order species were used in this study.

Behavioural & social sciences study design

All studies must disclose on these points even when the disclosure is negative.

Study description	N/A
Research sample	N/A

Sampling strategy	N/A
Data collection	N/A
Timing	N/A
Data exclusions	N/A
Non-participation	N/A
Randomization	N/A

Ecological, evolutionary & environmental sciences study design

All studies must disclose on these points even when the disclosure is negative.

Study description	N/A
Research sample	N/A
Sampling strategy	N/A
Data collection	N/A
Timing and spatial scale	N/A
Data exclusions	N/A
Reproducibility	N/A
Randomization	N/A
Blinding	N/A

Did the study involve field work? ☐ Yes ☒ No

Reporting for specific materials, systems and methods

We require information from authors about some types of materials, experimental systems and methods used in many studies. Here, indicate whether each material, system or method listed is relevant to your study. If you are not sure if a list item applies to your research, read the appropriate section before selecting a response.

Materials & experimental systems

n/a	Involved in the study	
☒	☐	Antibodies
☒	☐	Eukaryotic cell lines
☒	☐	Palaeontology and archaeology
☒	☐	Animals and other organisms
☒	☐	Clinical data
☒	☐	Dual use research of concern
☒	☐	Plants

Methods

n/a	Involved in the study	
☒	☐	ChIP-seq
☒	☐	Flow cytometry
☒	☐	MRI-based neuroimaging

Plants

Seed stocks	N/A
Novel plant genotypes	N/A
Authentication	N/A
